# Acute Myopericarditis in the Post COVID-19 Recovery Phase

**DOI:** 10.7759/cureus.11247

**Published:** 2020-10-29

**Authors:** Mark D Rivera-Morales, Robert Pell, Jose Rubero, Latha Ganti

**Affiliations:** 1 Emergency Medicine, University of Central Florida College of Medicine/ HCA Healthcare Graduate Medical Education Consortium, Orlando, USA; 2 Emergency Medicine, Emergency Medicine Residency Program of Greater Orlando, Orlando, USA; 3 Emergency Medicine, Osceola Regional Medical Center, Kissimmee, USA; 4 Emergency Medicine, University of Central Florida College of Medicine, Orlando, USA; 5 Emergency Medicine, University of Central Florida College of Medicine/HCA Healthcare Graduate Medical Education Consortium, Orlando, USA; 6 Emergency Medicine, Envision Physician Services, Plantation, USA; 7 Emergency Medicine, Polk County Fire Rescue, Bartow, USA; 8 Emergency Medicine, HCA Healthcare Graduate Medical Education Consortium Emergency Medicine Residency Program of Greater Orlando, Orlando, USA

**Keywords:** covid-19, acute myopericarditis

## Abstract

The COVID-19 viral infection, caused by the novel coronavirus severe acute respiratory syndrome coronavirus-2 (SARS-CoV-2), is a currently ongoing global pandemic that, as of mid-October, 2020, has resulted in more than 38.7 million confirmed cases globally and has caused more than 1.1 million fatalities. COVID-19 infection is associated with severe life threatening respiratory and cardiac complications such as acute respiratory distress syndrome (ARDS), pneumonia, shock, cardiac arrhythmias, myocardial infarction and heart failure, particularly in the acute infectious stage. Acute myopericarditis is another reported cardiac complication of COVID-19. Case reports have been limited in reporting the effects of COVID-19 in the post-symptomatic period.

In this article, we present a case of acute myopericarditis resulting 6 to 8 weeks after testing positive for COVID-19. Here we will breakdown the initial emergency department (ED) presentation, with particular attention to the electrocardiogram (ECG) findings of acute myopericarditis. This case, to the our best knowledge and after an extensive literature review, depicts the first case of myopericarditis in the post COVID-19 infection recovery phase.

## Introduction

The COVID-19 viral infection, caused by the novel coronavirus SARS-CoV-2, is an ongoing global pandemic that has resulted in more than 38.7 million confirmed cases globally and has caused more than 1.1 million deaths as of mid October 2020. The United States of America has the highest number of reported cases and deaths worldwide, with more than 8 million confirmed cases and 250,000 deaths as a result of the virus, according to the World Health Organization (WHO) statistics [1]. COVID-19 was first reported in Wuhan, a city in the Hubei Province of China, in December 2019. Since then, the virus has spread at an alarming rate to more than 200 countries and territories worldwide, reaching a global pandemic status.

SARS-CoV-2 is from the same coronavirus subfamily as Severe Acute Respiratory syndrome (SARS) and Middle East Respiratory Syndrome (MERS) virus. Although the infectivity and mortality are higher in COVID-19 when compared to SARS and MERS, the literature of COVID-19 is still limited. It is known the viral capsid of this viral subfamily has a functional receptor in the angiotensin converting enzyme-2 (ACE-2) receptors. These ACE-2 receptors have been shown to be expressed in lung alveolar cells, cardiac tissue, gastrointestinal epithelial cells and endothelial cells of arteries and veins, thereby explaining the wide array of pulmonary and extrapulmonary symptoms of this viral infection [2-5]. Studies have been reported about various life threatening respiratory and cardiac complications of COVID-19 such as ARDS, pneumonia, septic and cardiogenic shock, cardiac arrythmias, myocardial infarction and heart failure, particularly in the acute infectious stage [6,7]. However, case reports have been limited in reporting the effects of COVID-19 in the post-symptomatic period.

Acute myopericarditis is a reported cardiac complication of COVID-19 [8-10]. It is estimated that 7% of patients suffer from myocardial injury due to this infection [6]. The exact mechanisms of how SARS-CoV-2 cause myocardial injury are not yet clearly understood, but proposed pathophysiological mechanisms of injury include direct damage to the myocytes by the virus, an exaggerated cytokine response by type 1 and type 2 cells, and interferon mediated systemic inflammatory response, as well as an altered myocardial demand-supply ratio and coronary plaques rupture and thrombosis [11-12]. 

In this article, we present a case of myopericarditis occurring several weeks after testing positive for COVID-19 and having a 15 day ICU hospitalization due to acute respiratory failure. We review the initial ECG findings and explain why, based on these, the most likely diagnosis was acute myopericarditis. 

## Case presentation

This is a case of a 73-year-old Hispanic male with past medical history of hypertension, dyslipidemia, diabetes mellitus type 2, and former nicotine dependence. The patient presented to our ED brought in via Emergency Medical Services (EMS) as a ST elevation myocardial infarction (STEMI) alert. The patient was transferred to our emergency department (ED) from a nearby urgent care center, where he presented with complaints of chest pain. The patient stated that he woke up with midsternal chest pain, 3 hours prior to arrival. He described the chest pain as a pressure-like, non-radiating and constant, 7 out of 10 in severity initially on arrival to urgent care. He stated the pain was worsened by deep breathing and movement and that nothing alleviated it. He also endorsed feeling short of breath and having dyspnea on minimal exertion, for example, by walking a few steps. The patient denied palpitations, diaphoresis, nausea, vomiting, headache, dizziness, syncope, weakness, fever, chills, abdominal pain, or other complaints. 

The patient stated that approximately 6 to 8 weeks prior, he was admitted in another hospital and was in critical condition due to COVID-19 infection complicated by pneumonia. The patient stated that he was discharged from the hospital less than 1 month ago and that he was feeling well after discharge until waking up this day with chest pain.

**Figure 1:**
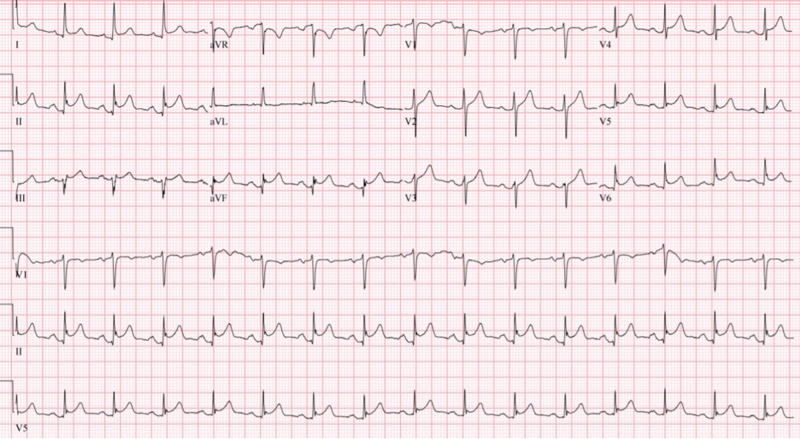
12-lead ECG done on arrival at the ED The rhythm is normal sinus rhythm. Note the concave shaped STE of different magnitudes in leads I, II, III, aVF, v2 to v6.

The initial vital signs were within normal limits except for heart rate fluctuating between 100 to 110 beats per minute. The patient reported the chest pain was still present, although it had slightly improved compared to the onset. The initial 12-lead electrocardiogram (ECG) in our ED is depicted below (figure 1).

The interventional cardiologist was consulted and upon evaluation of the ECG and because the patient was still experiencing chest pain, the decision was made to take the patient immediately for an emergent diagnostic left heart catheterization and coronary angiography. This study showed non obstructive coronary artery disease; thus no interventions were performed. A transthoracic echocardiogram was significant for a small pericardial effusion grade I diastolic dysfunction, with ejection fraction of 40% to 50% and increased wall thickness as well as concentric hypertrophy. Shortly after the coronary angiography, the serum troponin I levels came back within normal limits and the complete blood count was remarkable for a neutrophil-predominant leukocytosis (WBC of 20.63 K/mm^3^). Chest radiograph was negative for acute cardiopulmonary process. Given these findings, the diagnosis of acute myopericarditis was confirmed and the diagnosis of STEMI was ruled out.

## Discussion

The ECG findings in acute myopericarditis have been well studied and discussed in literature. The electrocardiographic abnormalities have been described in the past as evolving through four stages [13,14]. Stage I is usually the more obvious abnormality and represents the most common pattern seen in acute pericarditis. Stage I abnormalities include ST segment elevation (STE), prominent T waves, and PR segment depression. Stage II represents a resolution of the STE. Stage III involves T wave inversions that are usually in the distribution of the previously seen STE. Finally, stage IV represents a normalization of all initial ECG changes.

**Figure 2:**
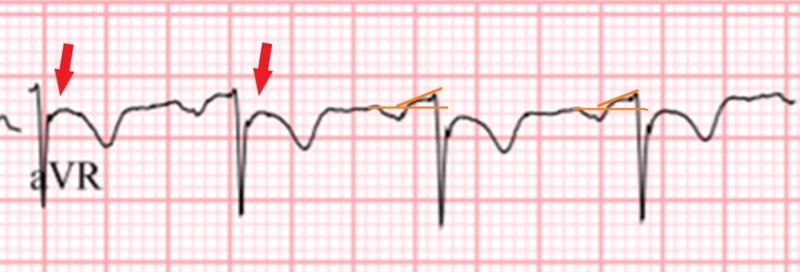
Close up of lead aVF showing the reciprocal changes in acute myopericarditis. Note the marked STD (red arrows), as well as the PR segment elevation (orange lines).

The STE in myopericarditis is usually widespread and expected to be present in all leads except leads aVF and I. STE is usually concave in shape and the magnitude of that STE is usually 2 to 4 mm. STE of magnitude of more than 5 mm or with a flat or convex shape is more suggestive of acute MI [15]. However, the degree of STE can vary in the different leads from prominent to very subtle depending if there is a focal inflammatory process occurring, like in the case of a focal myocarditis. This clinical picture represents a challenge to the physician who is expected to make a quick interpretation of that initial ECG, especially in the setting of a suspected STEMI. In the case presented, we believe this played a role in seeing more prominent STE in the inferior leads and that the STE in the rest of the leads were very subtle, less than 2mm in magnitude. The ECG of our patient does present concave shaped STE in leads I, II, aVF, v2 to v6 of different magnitudes, with reciprocal ST depression (STD) seen in lead aVR (Figure 2).

**Figure 3:**
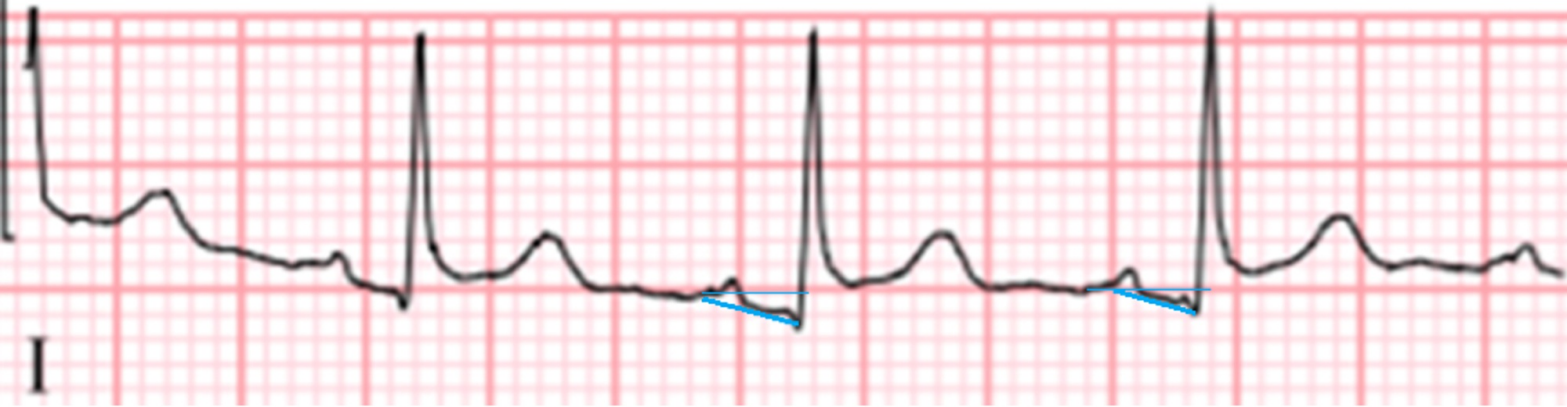
Close up of lead 1 showing PR segment depression (blue lines).

PR segment depression, also seen in our patient’s ECG, is an ECG finding suggestive of atrial inflammation and irritation, a feature that is highly suggestive of stage I myopericarditis (Figure 3) [14].

**Figure 4:**
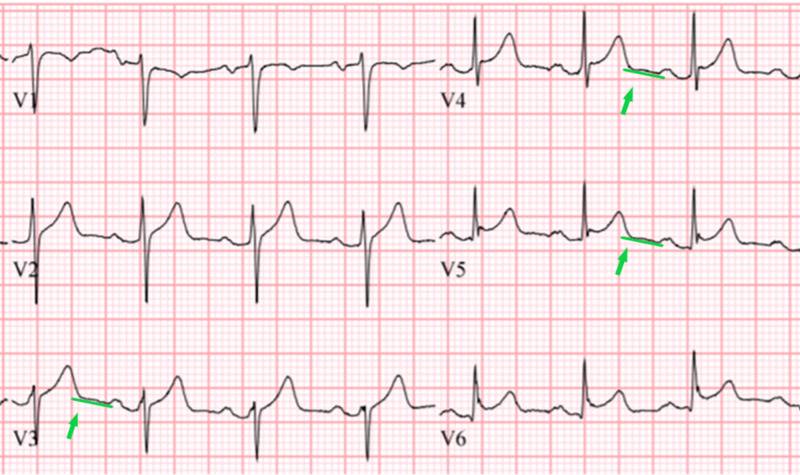
Close up of precordial leads showing Spodick’s sign, or downsloping TP segments (green lines and arrows in leads v3 to v5)

It is seen more prominently in the inferior and lateral leads. In the rightward-pointing axis leads aVF and v1, reciprocal PR segment elevation (figure 2) can be seen and sometimes it is more prominent than the PR segment depression itself [13]. Spodick’s sign, a downsloping TP segment, is another finding highly suggestive of pericardial inflammation and stage I myopericarditis. It is present in about 80% of patients with acute pericarditis and is an important finding when differentiating the diagnosis from ACS [16,17]. The sign is often best visualized in lead II and lateral precordial leads (Figure 4).

In addition, Spodick’s sign may also serve as an important distinguishing electrocardiographic tool between the acute pericarditis and acute coronary syndrome. In our case, the presence of PR segment depression as well as reciprocal PR segment elevation, in addition to Spodick’s sign, made the diagnosis of acute myopericarditis highly probable. 

Limitations of this case report include that we did not obtain histological confirmation of myocarditis and/or isolation of the SARS-CoV2 viral genome in the myocardium. For this we would have needed an endomyocardial biopsy, and such an invasive procedure was not indicated in this case.

The diagnosis of acute myopericarditis should always be considered when a patient presents with chest pain and suspected STEMI. There are many cardiotropic viruses that are well known to cause pericardial and myocardial injury such as influenza virus, enterovirus, or parvovirus and now SARS-CoV-2. When considering myopericarditis, the clinician should always consider a viral etiology, especially in the post-viral infection period. Meticulous analysis of the 12 lead ECG in suspected myopericarditis, placing particular attention to the ECG findings mentioned above is important in order to avoid delays in diagnosis and treatment, including exposing the patient to an unnecessary cardiac catheterization. Nonetheless, the false-positive cardiac catheterization laboratory activation for a suspected STEMI is relatively common in clinical practice, especially in the era of emphasis on rapid time to reperfusion therapy metrics [18].

## Conclusions

This case report describes myopericarditis in a patient that has recovered from COVID-19 pneumonia several weeks prior. This case report contributes to the currently evolving COVID-19 literature and describes yet another complication of this infection, and our evolving understanding of it.
